# Editorial: Advances in iPSC technology for disease modeling and therapeutic applications

**DOI:** 10.3389/fcell.2023.1261279

**Published:** 2023-07-31

**Authors:** Methichit Wattanapanitch, Thanathom Chailangkarn, Helen Cristina Miranda, Alysson Renato Muotri

**Affiliations:** ^1^ Siriraj Center for Regenerative Medicine, Faculty of Medicine Siriraj Hospital, Mahidol University, Bangkok, Thailand; ^2^ Virology and Cell Technology Research Team, National Center for Genetic Engineering and Biotechnology (BIOTEC), National Science and Technology Development Agency (NSTDA), Pathum Thani, Thailand; ^3^ Department of Genetics and Genome Sciences, Case Western Reserve University, Cleveland, OH, United States; ^4^ Department of Pediatrics, School of Medicine, University of California San Diego, La Jolla, CA, United States; ^5^ Department of Cellular and Molecular Medicine, School of Medicine, University of California San Diego, La Jolla, CA, United States; ^6^ Center for Academic Research and Training in Anthropogeny (CARTA), Kavli Institute for Brain and Mind, Archealization Center (ArchC), La Jolla, CA, United States

**Keywords:** induced pluripotent stem cells, disease modeling, cell-based therapy, regenerative medicine, organoids, drug screening, differentiation

Over the past decade, substantial scientific progress has been made using induced pluripotent stem cells (iPSCs). Disease-specific iPSC lines can be generated from the patient’s somatic cells. With the recent advances in developmental biology, next-generation sequencing, single-cell analysis, three-dimensional (3D) culture, tissue engineering, and genome editing technology, large quantities of disease-relevant cell types that are otherwise inaccessible can be produced in a dish. Such platforms provide a powerful model for studying disease pathology and drug screening. This Research Topic, **“** *Advances in iPSC technology for disease modeling and therapeutic applications*,” published in Frontiers in Cell and Developmental Biology, presents recent advances in iPSCs in disease modeling and regenerative medicine, which allow a better understanding of underlying pathological mechanisms and the development of novel therapeutic approaches.

Guided differentiation of iPSCs into tissue-specific cell types requires specific signals recapitulating those in fetal development. Recently, 3D organoid culture system has revolutionized the field by mimicking the organization and functionality of organs in a dish, facilitating the study of developmental and cellular processes (Clevers, 2016). In this Research Topic, Asal et al. developed a protocol to generate functional lacrimal grand organoids from human iPSC-derived multizonal ocular cells. The lacrimal gland is a tubuloacinar exocrine gland consisting of acinar, ductal, and myoepithelial cells, which play an essential role in tear production. Dysfunction of the lacrimal gland causes dry eye, poor ocular surface maintenance, and a risk of infection. In the past decades, several approaches have been developed to generate lacrimal glands using various cell types, including embryonic and adult stem cells. In this study, the authors improved the differentiation protocol for generating functional lacrimal gland organoids capable of secreting tear components upon stimulation. Comprehensive metabolomic profiling confirmed the differentiation and maturation toward lacrimal phenotype. The approach provides a versatile platform for investigating genetic diseases associated with the lacrimal gland and developing novel therapeutic agents.

In spite of the advances in the generation of hiPSC-derived organoids, single-cell types of interest are still desired for solving specific questions. One such cell type is stellate cells, a prime neuron subtype in the medial entorhinal cortex (MEC) playing a vital role in spatial memory processing. In Alzheimer’s disease (AD) context, they were reportedly the first population in such brain area to accumulate Amyloid beta, which could presumably be associated with spatial processing perturbation in early AD. Bergmann et al. developed a directed differentiation protocol of hiPSCs into putative stellate cells from the ground up. Stellate cell-like cells were generated by overexpression of a set of selected transcription factors in hiPSCs, previously identified through single-cell RNA-sequencing of developing and postnatal porcine medial entorhinal cortex. These cells could be further used as a relevant model for AD study.

HiPSC technology was also used for investigating mechanisms regulating heart regeneration. Upon cardiac injury leading to the death of cardiomyocytes, mature epicardial cells transformed into epicardium-derived progenitor cells, which could give rise to new cardiomyocytes. Such transformation has been suggested to be regulated by the brain. Wasserman et al. demonstrated that oxytocin (OXT), a neuropeptide hormone produced and released by the hypothalamus and pituitary gland, respectively, promotes the induction of mature-like hiPSC-derived epicardial cells into epicardial progenitors. Using the zebrafish model, the authors also showed increased OXT expression in the brain and heart regeneration after the cardiac injury. These findings shed light on translational applications for restoring damaged heart tissue.

Apart from cardiac injury modeling, iPSC platforms have also been used to model various cardiovascular diseases. Bissoli et al. summarized current limitations in the field and future promising avenues. While iPSC-derived cardiomyocytes (iPSC-CMs) have provided a powerful tool for creating a “heart in a dish,” researchers face a significant challenge in that the iPSC-CMs often exhibit an immature phenotype and do not recapitulate several features of adult CMs. Continuous efforts in tissue engineering, cardiac organoid, and microfluidic heart-on-a-chip platforms are being made to improve the maturity and functionality of iPSC-CMs.

Several neurological diseases have been studied using iPSC technology as well (Jovanovich et al., 2021). One of them is amyotrophic lateral sclerosis (ALS), a devastating neurodegenerative disease affecting motor neurons (MNs). Amoros et al. discussed the current methods to generate MNs, including direct conversion from patients’ somatic cells and differentiation from patient-derived iPSCs, emphasizing the need for standardized MN differentiation protocols to reduce the variation among studies and, therefore, allow for cross-study interpretation. Such requirement is critical for drug screening platforms. The authors also reviewed the advances of using 3D cultures and bioinformatics in ALS research, which would potentially accelerate the discovery of novel therapeutical targets and treatments.

The hiPSCs technology also gives hope to hair loss patients worldwide due to its potential to regenerate hair follicles (HFs), essential structures within our skin responsible for hair growth. Vatanashevanopakorn et al. described HF biology and reviewed different sources for HF regeneration, including primary hair follicular cells and iPSCs, as well as their advantages and limitations. The authors also discussed the iPSC-based protocols to generate both single HF components and entire HFs, which, while advanced, still need better characterization before clinical utilization.

Finally, the hiPSC technology takes part in infectious disease research that relies heavily on animal models. While establishing significant knowledge, including pathogenesis, transmission, and host responses to pathogens, animal models still have some limitations, such as the species-specific difference in physiology, immune responses, and susceptibility to the pathogens. This is the case for SARS-CoV-2 (Larijani et al., 2021), the virus responsible for causing the global COVID-19 pandemic which started in December 2019. Given the ability to differentiate into all somatic cell types, hiPSCs provide a powerful tool for studying viral infection and developing novel therapy. Karami et al. described the applications of iPSCs in the study and treatment of COVID-19. The authors highlighted various iPSC-derived cells used to study SAR-CoV-2 infection and screened for FDA-approved drugs against viral infection. Modeling COVID-19 using iPSC-derived cells enables a better understanding of pathogenic mechanisms, leading to the discovery of new therapies.
